# Supplementary material for: A UMLS-based spell checker for natural language processing in vaccine safety
Source: BMC Med Inform Decis Mak. 2007 Feb 12;7:3. doi: 10.1186/1472-6947-7-3 (PMC1805499; doi:10.1186/1472-6947-7-3)
Supplement: Additional file 13 — RAP application directory. Sets up directory for RAP (RDF application for PHP) source code [file 1472-6947-7-3-S13.gz › rap/test/test_RdqlQuery.php]

Test Traverse MemModel

php
define("RDFAPI\_INCLUDE\_DIR", "./../api/");
include(RDFAPI\_INCLUDE\_DIR . "RdfAPI.php");
// Filename of an RDF document
$base="employees.rdf";
// Create a new MemModel
$model = new MemModel();
// Load and parse document
$model-load($base);
// Show input model
//$model->writeAsHtmlTable();
echo "

";
echo "  
show the age of all employees ,using the RdqlResultIterator  
";
$rdql\_query = '
SELECT ?givenName, ?age
WHERE (?x, v:age, ?age),
(?x, vcard:N, ?blank),
(?blank, vcard:Given, ?givenName)
USING vcard FOR 
v FOR ';
//query model, get RDQLResultIterator
$rdqlIter = $model->rdqlQueryasIterator($rdql\_query);
//get different Result labels as array
$result\_labels=$rdqlIter->getResultLabels();
echo "  
**Result Label Names:** ";
for ($i=0; $i **Number of results:** ".$rdqlIter->countResults();
echo "  
**Result objects, serialized to string:**  
";
//serialize result to string
while ($rdqlIter->hasNext()) {
$curren\_result=$rdqlIter->next();
echo "  
";
for ($j=0; $j toString()."  
 ";
};
echo "

";
// show emails of all employees
$rdql\_query = '
SELECT ?givenName, ?familyName, ?email
WHERE (?person vcard:N ?blank1)
(?blank1 vcard:Given ?givenName)
(?blank1 vcard:Family ?familyName)
(?person vcard:EMAIL ?blank2)
(?blank2 rdf:value ?email)
AND ?person ~~ "/example.com\/EMPLOYEES\//i"
USING vcard FOR 
v FOR ';
$res = $model->rdqlQuery($rdql\_query);
RdqlEngine::writeQueryResultAsHtmlTable($res);
echo "  
show all employees over 30   
";
// show all employees over 30
$rdql\_query = '
SELECT ?employee, ?age
WHERE (?x, vcard:FN, ?employee), (?x, , ?age)
AND ?age > 30
USING vcard FOR 
v FOR ';
$res = $model->rdqlQuery($rdql\_query);
RdqlEngine::writeQueryResultAsHtmlTable($res);
echo "

";
// find out whose number is it: +1 111 2212 431
$rdql\_query = '
SELECT ?x, ?type
WHERE (?x, vcard:TEL, ?tel),
(?tel, rdf:value, ?telNumber)
(?tel, rdf:type, ?type)
AND ?telNumber eq "+1 111 2212 431"
USING vcard FOR 
v FOR ';
$res = $model->rdqlQuery($rdql\_query);
RdqlEngine::writeQueryResultAsHtmlTable($res);
echo "

";
// show office telephone numbers of all employees
$rdql\_query = '
SELECT ?givenName, ?familyName, ?telNumber
WHERE (?person vcard:N ?blank1)
(?blank1 vcard:Given ?givenName)
(?blank1 vcard:Family ?familyName)
(?person vcard:TEL ?blank2)
(?blank2 rdf:value ?telNumber)
(?blank2 rdf:type ?type)
AND ?person ~~ "/example.com\/EMPLOYEES\//i" && ?type eq vcard:work
USING vcard FOR 
v FOR ';
$res = $model->rdqlQuery($rdql\_query);
RdqlEngine::writeQueryResultAsHtmlTable($res);
?>
